# PI3K/Akt signaling pathway is essential for de novo hair follicle regeneration

**DOI:** 10.1186/s13287-020-01650-6

**Published:** 2020-04-03

**Authors:** Yu Chen, Zhimeng Fan, Xiaoxiao Wang, Miaohua Mo, Shu Bin Zeng, Ren-He Xu, Xusheng Wang, Yaojiong Wu

**Affiliations:** 1 State Key Laboratory of Chemical Oncogenomics, and Shenzhen Key Laboratory of Health Sciences and Technology, Tsinghua Shenzhen International Graduate School, Tsinghua University, Shenzhen, China; 2 Tsinghua-Berkeley Shenzhen Institute (TBSI), Tsinghua University, Shenzhen, China; 3 Faculty of Health Sciences, University of Macau, Macau, China; 4 School of Pharmaceutical Sciences (Shenzhen), Sun Yat-sen University, Guangzhou, China

**Keywords:** Epi-SCs, SKPs, Hair follicle regeneration, PI3K-Akt signal

## Abstract

**Background:**

Cultured epidermal stem cells (Epi-SCs) and skin-derived precursors (SKPs) were capable of reconstituting functional hair follicles after implantation, while the signaling pathways that regulate neogenic hair follicle formation are poorly investigated. In this study, we aimed to understand the interactions between Epi-SCs and SKPs during skin organoid formation and to uncover key signal pathways crucial for de novo hair follicle regeneration.

**Methods:**

To track their fate after transplantation, Epi-SCs derived from neonatal C57BL/6 mice were labeled with tdTomato, and SKPs were isolated from neonatal C57BL/6/GFP mice. A mixture of Epi-SCs-tdTomato and SKPs-EGFP in Matrigel was observed under two-photon microscope in culture and after implantation into excisional wounds in nude mice, to observe dynamic migrations of the cells during hair follicle morphogenesis. Signaling communications between the two cell populations were examined by RNA-Seq analysis. Potential signaling pathways revealed by the analysis were validated by targeting the pathways using specific inhibitors to observe a functional loss in de novo hair follicle formation.

**Results:**

Two-photon microscopy analysis indicated that when Epi-SCs and SKPs were mixed in Matrigel and cultured, they underwent dynamic migrations resulting in the formation of a bilayer skin-like structure (skin organoid), where Epi-SCs positioned themselves in the outer layer; when the mixture of Epi-SCs and SKPs was grafted into excisional wounds in nude mice, a bilayer structure resembling the epidermis and the dermis formed at the 5th day, and de novo hair follicles generated subsequently. RNA-Seq analysis of the two cell types after incubation in mixture revealed dramatic alterations in gene transcriptome, where PI3K-Akt signaling pathway in Epi-SCs was significantly upregulated; meanwhile, elevated expressions of several growth factors and cytokine potentially activating PI3K were found in SKPs, suggesting active reciprocal communications between them. In addition, inhibition of PI3K or Akt by specific inhibitors markedly suppressed the hair follicle regeneration mediated by Epi-SCs and SKPs.

**Conclusions:**

Our data indicate that the PI3K-Akt signaling pathway plays a crucial role in de novo hair follicle regeneration, and the finding may suggest potential therapeutic applications in enhancing hair regeneration.

## Introduction

Adult mouse and human skin consist of a keratinized-stratified epidermis and an underlying layer of the dermis [1]. The hair follicles, sebaceous glands, and sweat glands, which extend deep into the dermis, are derived from a single layer of multipotent progenitors during skin development and hair follicle morphogenesis [2–4]. Embryonic hair follicle development starts with the formation of placode in the early epidermis and followed by dermal-condensation [5]. Wnt signal has been considered as the earliest event and plays a predominant role in hair follicle morphogenesis [6]. In de novo hair follicle regeneration in mice, Wnt signaling pathway is also considered essential [7]. Besides embryonic hair follicle development, the hair follicle could be rebuilt with isolated hair follicle stem cells or cultured epidermal stem cells (Epi-SCs) in combination with dermal papilla (DP) cells or skin-derived precursors (SKPs) [8–10]. Despite the molecular mechanisms regulating the cyclic regeneration of the hair follicle have been intensively studied [11–13], the signaling pathways mediating hair follicle de novo regeneration have been poorly investigated.

In this study, we aimed to understand the communications between Epi-SCs and SKPs during skin organoid formation and to uncover signaling pathways crucial for de novo hair follicle regeneration. We found dynamic motions of Epi-SCs and SKPs in de novo hair follicle formation, uncovered marked gene transcriptional changes in the cells, and identified a crucial role of PI3K/Akt signaling pathway in de novo hair follicle regeneration.

## Materials and methods

### Mice

Five- and six-week old BALB/c nu/nu mice and 7-week-old C57BL/6 mice were purchased from the Guangdong Medical Laboratory Animal Center, Guangzhou, China. Six-week-old C57BL/6/GFP mice were derived from the Cyagen BioSciences, Guangzhou, China. These mice were maintained in a temperature-controlled environment (20 ± 1 °C). All animal procedures were performed with the approval of the Animal Ethics Committee of Tsinghua Shenzhen International Graduate School.

### Isolation and culture of Epi-SCs and SKPs

Full-thickness dorsal skin tissue was collected from C57BL/6 or C57BL/GFP mice 0~72 h after birth. The tissue was washed 3 times in PBS, cut into 2~3 mm^2^ pieces, and digested with 0.35% Dispase II (sigma) for 40 min at 37 °C. The epithelial layer was removed manually. Epi-SCs were isolated on account of their high-adhesive property as described previously [8, 14]. Briefly, the epidermis was cut into pieces, which were treated with 0.035% collagenase I (Sigma) at 37 °C for 1 h with shaking gently and filtered with a 40-μm cell strainer. The cells were seeded in tissue culture dishes and cultured in CnT-07 PCT Epidermal Keratinocyte Medium (CELLnTEC Advanced Cell Systems). The non-adherent cells were removed and the adherent cells (Epi-SCs) were maintained. When reaching 70–80% confluence, the cells were digested with accutase (Sigma) and subcultured. SKPs were prepared as previously described [8, 15]. Briefly, single dermal cells derived from the dermal tissue were incubated in a 10-cm non-treated dish in 10 ml Dulbecco’s modified Eagle’s medium (DMEM)/F12, 3:1 (Gibco) containing B27 (Gibco), 20 ng/ml epidermal growth factor (EGF, Peprotech), and 40 ng/ml basal fibroblast growth factor (bFGF, Peprotech) and incubated in a 37 °C, 5% CO_2_ tissue culture incubator.

### Cell labeling and in vitro cell tracing

Epi-SCs were transduced with tdTomato by retroviruses (Epi-SCs-tdTomato). 10^6^ Epi-SCs seeded in a 10-cm tissue culture dish were infected with tdTomato by retroviruses (MOI = 10) in the presence of 5 μg/mL polybrene (Sigma) in the culture medium. The medium was replaced by regular growth medium after 12 h. The cells were examined for tdTomato expression at 48 h and subjected to transplantation. 1 × 10^6^ Epi-SCs-tdTomato and 2 × 10^6^ SKPs-EGFP were mixed in Matrigel, seeded in a 33-mm confocal dish, incubated at 37 °C for 15 min, and then added with 2 ml DMEM/F12 3:1. Live-cell images were recorded for 36 h at 37 °C and 5% CO_2_ on Leica DMI6000 confocal live-cell imaging system.

### Cell sorting and RNA-Seq

Epi-SCs-tdTomato and SKPs-EGFP were cultured in Matrigel individually or in mixture for 24 h. Then, cells were recovered from the matrix. Epi-SCs-tdTomato and SKPs-EGFP were separated through cell sorting by a flow cytometer (Becton Dickinson). Total RNA was extracted from the Epi-SCs and SKPs using Trizol (TAKARA) according to the manufacturer’s instructions, and libraries were constructed using VAHTS mRNA-seq V3 Library Prep Kit for Illumina® (Vazyme). The qualified libraries were used for sequencing using Illumina HiSeqTM 2500 by Gene Denovo. All raw data of RNA sequencing were corresponded to the mouse genome using TopHat V2.0.3 and Bowtie2. Gene expression was measured by FPKM (fragment per kilobase of transcript per million mapped reads), which computed with Cufflinks V2.1.1. Enricher was used to analyze gene ontology enrichment. And heatmaps were carried out by GO analysis, coupled with the KEGG pathways database. In addition, differentially expressed genes (DEGs) were identified by edgeR analysis with FDR < 0.05.

### Cell transplantation and hair follicle regeneration

BALB/c nu/nu mice (4–5 weeks old) were anesthetized with sodium pentobarbital (50 mg/kg). Symmetrical full-thickness skin wounds were created on the back with a 2-mm-diameter skin biopsy punch as previously described [16]. 1 × 10^6^ Epi-SCs-tdTomato were mixed with 2 × 10^6^ SKPs-EGFP and encapsulated in 20 μl Matrigel (BD BioSciences). The cell-Matrigel was incubated at 37 °C for 30 min and implanted into an excisional wound. The wound was then covered with Tegaderm (3M) transparent dressing and self-adhering elastic bandage successively. Three weeks later, mice were sacrificed and wound tissue samples were obtained for histological analysis. In PI3K/Akt inhibitor treatment, Perifosine (Akt inhibitor) and LY294002 (PI3K inhibitor) were added into the cell mixture, respectively, after dilution with Matrigel, resulting in a final concentration of 2 mM Perifosine or 500 μM LY294002 in the graft. The mixture was incubated in a tissue culture incubator for 30 min and then implanted into wounds in mice.

### Two-photon microscopy

Movement of Epi-SCs-tdTomato and SKPs-EGFP after implantation into skin wounds were tracked by two-photon microscopy according to a method previously described [17]. Mice were anesthetized with isoflurane and fixed on the platform of the microscope. A custom tweezer was placed to fix the skin wound. Images were recorded with a FV300 Olympus two-photon microscopy. Laser beam was 940 nm for GFP and 1040 nm for tdTomato. Serial optical sections were obtained in 5 μm steps to image a total depth of about 300 μm of tissue.

### Immunofluorescence staining

Dorsal skin tissues of nu/nu mice were harvested and fixed with 4% paraformaldehyde (PFA, Sigma), washed with PBS, and dehydrated with 30% sucrose successively. Tissues were embedded in OCT and sectioned (10-μm thickness). Samples were washed with PBS and blocked with 3% BSA/PBS containing 0.2% Triton X-100 (Sigma) at 37 °C for 1 h. Samples were incubated with primary antibodies in 1% BSA/PBS at appropriate concentrations at 4 °C overnight: CD49f-biotin (BioLegend, 1:150), K14 (BioLegend, 1:100), K1 (BioLegend, 1:100), and nestin (Santa Cruz, 1:100). Samples were washed with PBS and detected with fluorescence-conjugated secondary antibodies. Nuclei were stained with DAPI. Samples were examined under confocal laser scanning microscope (FV1000, Olympus, Japan).

## Results

### Tracing of Epi-SCs and SKPs in hair follicle genesis

**Fig. 1:**
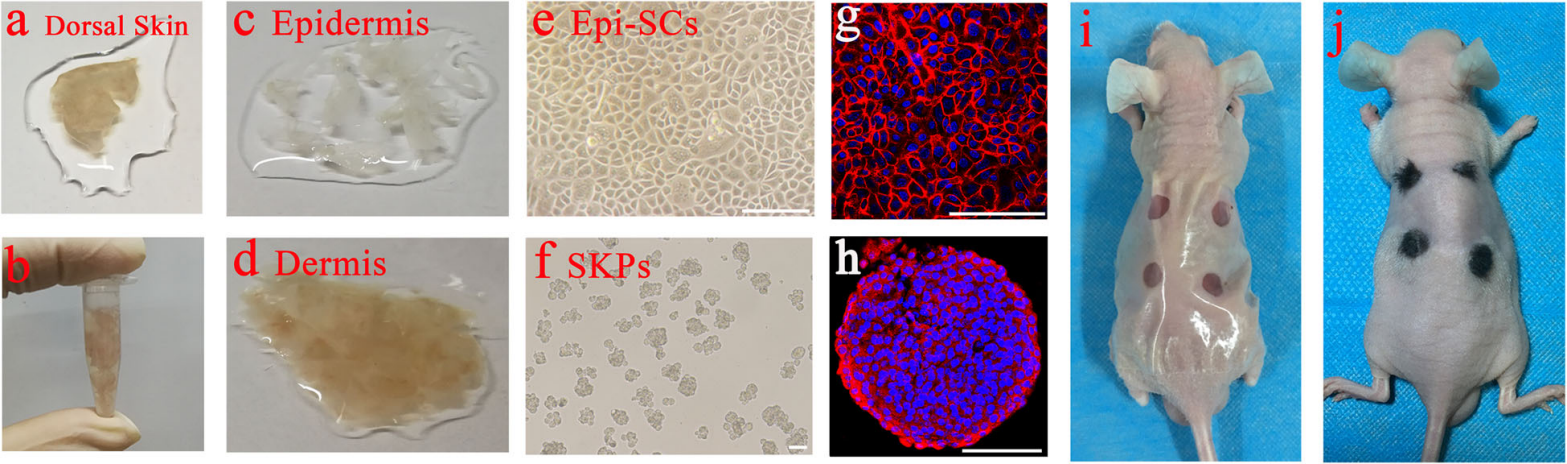
A schematic diagram of Epi-SCs and SKP transplantation for hair follicle regeneration. Dorsal skin tissue in full thickness was collected from neonatal C57 mice (**a**), which was cut into pieces (**b**). The tissue was separated into the epidermis and dermis after treatment with dispase II (**c**, **d**). Epidermal stem cells (Epi-SCs) were derived from the epidermis as described in the “Methods” section, which grew in monolayer (**e**) and expressed CD49f as detected by immunofluorescence staining (red, **g**). Skin-derived precursors (SKPs) were derived from the dermis as described in the “Methods” section, which were grown in spheroids and expressed nestin as detected by immunofluorescence staining (red, **h**). Full thickness excisional skin wounds were prepared in nu/nu mice (**i**), and a mixture of Epi-SCs and SKPs in Matrigel was implanted into the wound, which resulted in the growth of black hairs (**j**). Scale bar, 50 μm

**Fig. 2:**
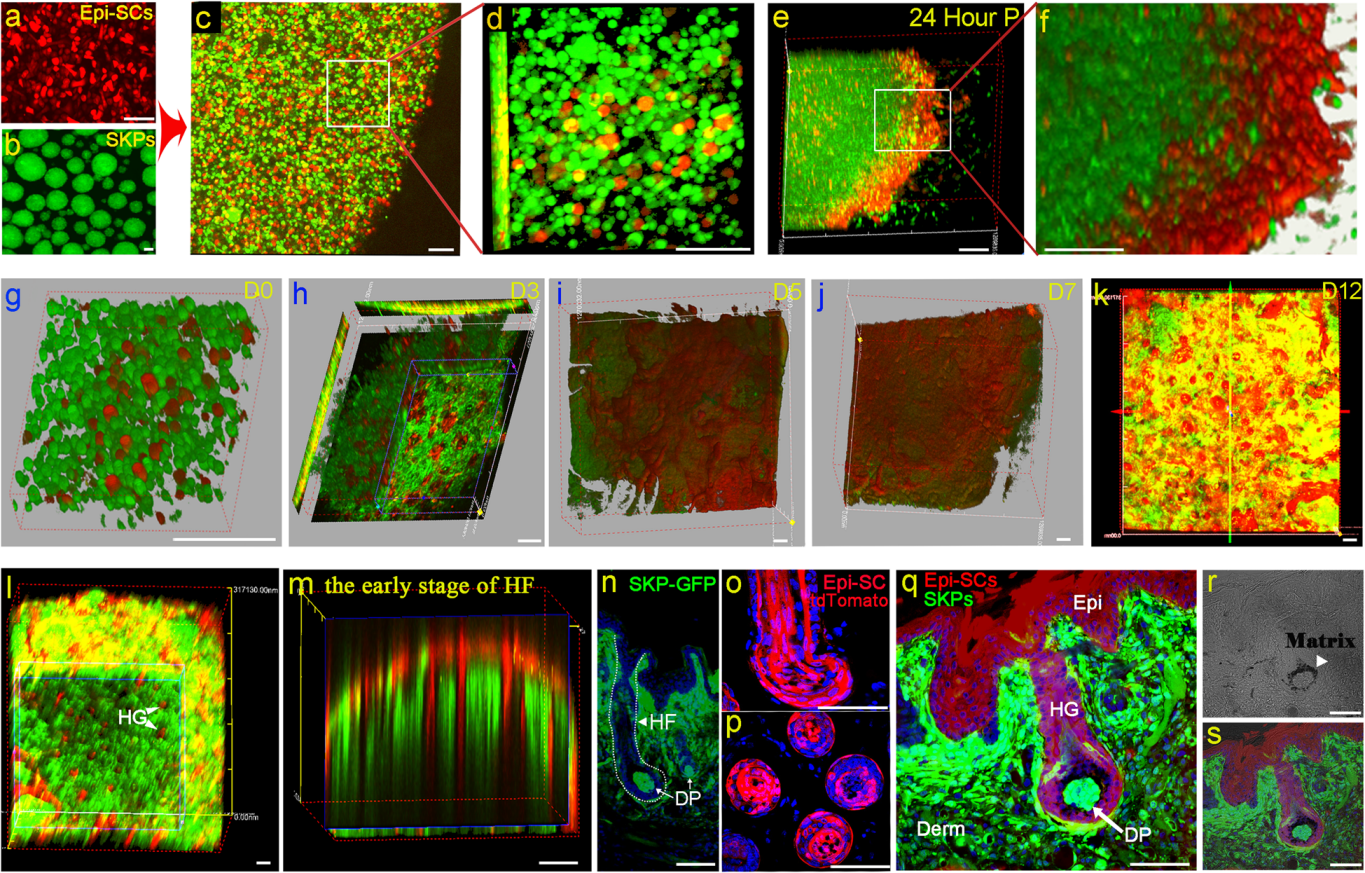
Fates of Epi-SCs and SKPs in mixture in vitro and in vivo. **a**–**f** Epi-SCs and SKPs in skin organoid formation*.* Epi-SCs labeled with tdTomato (red) were cultured in monolayer (**a**) and SKPs derived from C57-EGFP mice were grown in spheroids (**b**). Single cells of the above were mixed evenly in Matrigel (**c**, **d**) to form a sphere, which was incubated at 37 °C for 24 h. Cross-sections of the sphere showed that the cells were repopulated into two compartments, with the Epi-SCs-tdTomato in the outer layer (red) and the SKPs-EGFP (green) in the inner compartment (**e**, **f**), resembling the structure of bilayer skin. **g**–**r** Fates of Epi-SCs and SKPs in vivo. A mixture of single Epi-SCs-tdTomato and SKPs-EGFP in Matrigel was implanted into an excisional wound in a nude mouse, and the graft was observed under two-photon microscope. In the first 3 days, Epi-SCs aggregated forming spheres (red, **g** and **h**). By day 5, Epi-SCs migrated upward and formed an epidermis-like layer over SKPs (**i**, **j**). Some Epi-SCs in the layer then moved downward into the SKPs forming a primary structure of the hair follicle by 12 days; images of graft surface (**k**), horizontal section (**l**), and vertical section (**m**) were shown. **n**–**q** Tissue sections of the wound at 14 days post transplantation showed that the Epi-SCs and SKPs formed de novo skin structures, where the DPs and dermal cells were derived from the GFP-expressing SKPs (**n**, **q**); the epidermis and the trunk of the hair follicle were formed by the Epi-SCs-tdTomato (**o**, **q**). **r**, **s** At the interface of DP and follicle germ was the matrix (**r**, **s**). HF, hair follicle; Epi, epidermis; DP, dermal papilla. Scale bar, 100 μm


Transplantation of a mixture of Epi-SCs and SKPs into excisional wounds in nude mice could form de novo hair follicles (illustrated in Fig. 1) as previously reported by our group [8]. To understand the process of the hair follicle formation, we performed two-photon microscopic observation of the fates of Epi-SCs and SKPs in the graft. For cell tracing, Epi-SCs, which expressed typical marker CD49f (Fig. 1g), and SKPs, which expressed nestin as detected by immunofluorescence staining (Fig. 1h), were labeled with tdTomato and EGFP, respectively, before transplantation (Fig. 2a, b). Single Epi-SCs-tdTomato and SKPs-EGFP were mixed evenly in Matrigel (Fig. 2c, d), incubated in a tissue culture chamber, and monitored under a confocal microscope with a video camera for 24 h. We observed dynamic movements of the cells and structural re-organization to form skin organoid, with Epi-SCs migrating outward forming an epidermis-like layer on the surface, and SKPs gathering underneath the Epi-SCs layer forming the “dermis” (Fig. 2e, f, and Video 1), resulting in the formation of a bilayer structure, similar to the skin. It appeared that Epi-SCs and SKPs were intrinsically programmed to migrate and position. To examine whether this phenomenon occurred in vivo, we implanted the mixture of Epi-SCs-tdTomato and SKPs-EGFP in Matrigel into excisional wounds in nude mice and tracked the fate of the cells under two-photon microscope. In day 0, cells remained in individuals in the matrix (Fig. 2g); at day 3, Epi-SCs aggregated and formed many spheres (Fig. 2h); and at day 5, Epi-SCs were found to form a layer on the surface of wound bed and SKPs were underneath (Fig. 2i, j). Intriguingly, at day 12 some Epi-SCs in the “epidermis” migrated downward and penetrated deep to the SKP layer, forming a primary structure of the hair follicle (Fig. 2k–m). Histological analysis of the wound at day 14 showed that the SKPs formed the DP in neogenic hair follicles and abundant dermal cells in the dermis (Fig. 2q, n), and the Epi-SCs formed the epidermis and the trunk of the hair follicle (Fig. 2o–q). Interestingly, at the interface of DP and bulb of the follicle, the matrix was observed (Fig. 2r, s), which as an outcome of interactions between the two stem cell populations would generate hair shaft subsequently.

**Additional file 1: Video 1.** Tracking of Epi-SCs and SKPs in skin organoid formation. Epi-SCs and SKPs labeled with tdTomato (red) and EGFP (green), respectively, were mixed evenly in Matrigel to form a 3D sphere. The cells were incubated in a chamber at 37 °C and 5% CO_2_ and monitored under a confocal microscope. Live-cell images were captured at a time interval of 20 min. Epi-SCs moved dynamically outwards to the surface forming a layer similar to the epidermis, while the SKPs distributed underneath the Epi-SCs layer.


### PI3K/Akt signal is essential for de novo hair follicle regeneration

**Fig. 3:**
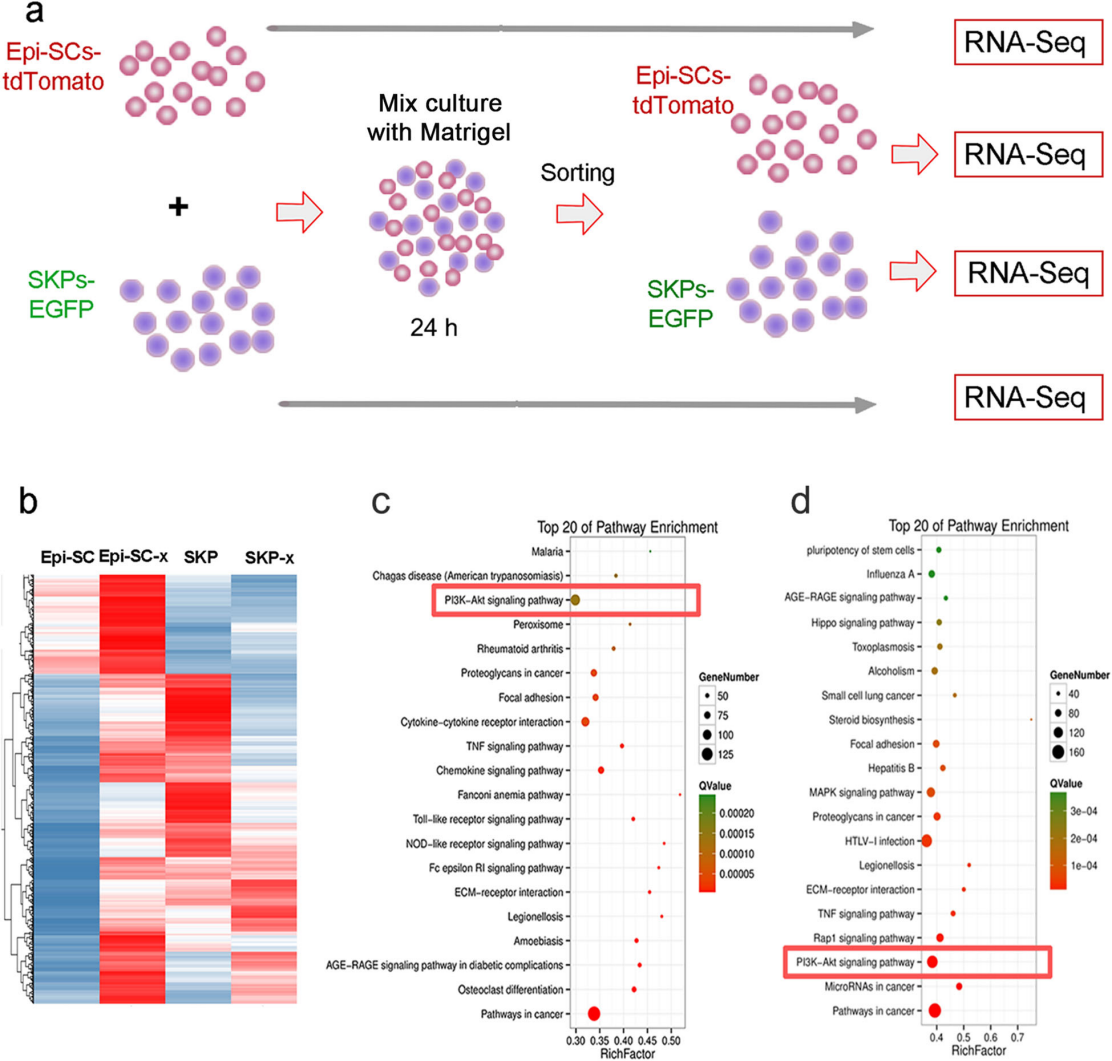
RNA-Seq of Epi-SC and SKPs. **a** A schematic diagram depicting sample preparation for RNA-Seq. Total RNA was extracted from Epi-SCs-tdTomato and SKPs-EGFP which were cultured separately or in mixture in Matrigel for 24 h. Cells were recovered from the matrix and separated by cell sorting using a flow cytometer. Total RNA was extracted from the cells and subjected to RNA-Seq analysis. **b** Transcriptome shows different gene transcriptional patterns of Epi-SCs and SKPs when cultured alone or in mixture (x). **c**, **d** KEGG analysis revealed active signals in Epi-SCs and SKPs when cultured in mixture, among them there was PI3K/Akt signal

**Fig. 4:**
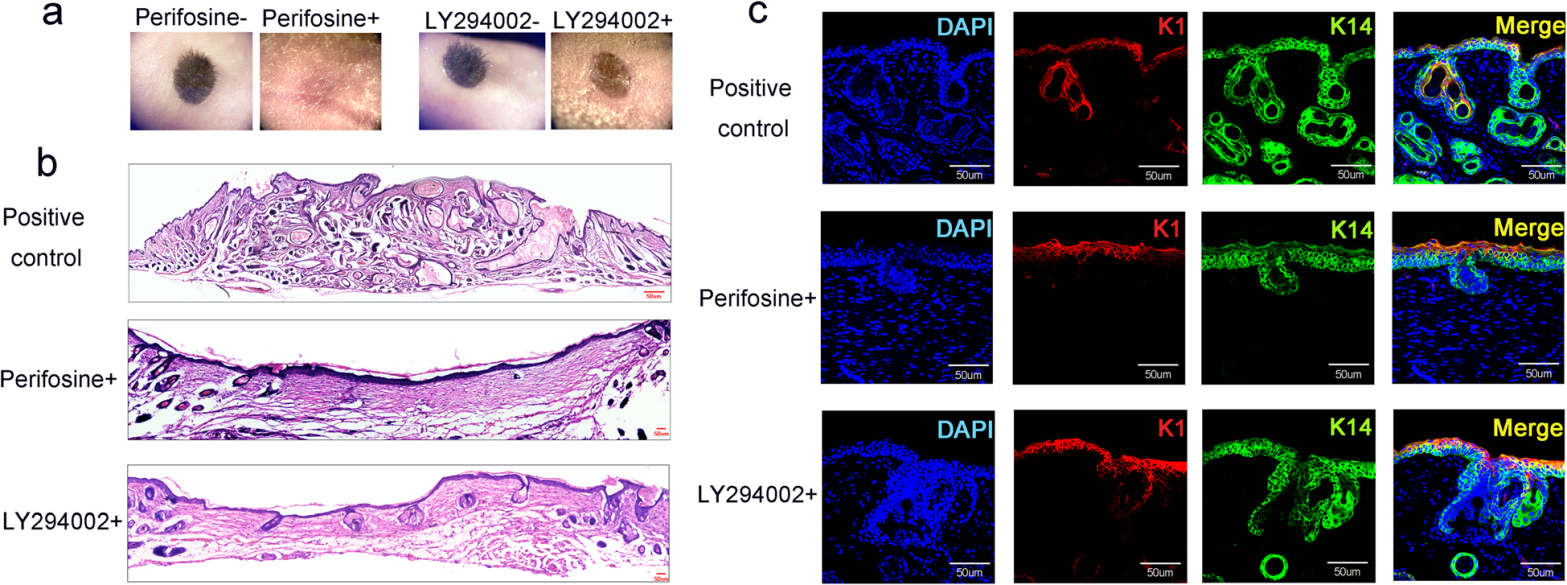
Analysis of PI3K/Akt inhibition on hair follicle regeneration. **a** Hair follicle reconstitution assay showed that addition of Periforsine or LY294002 to Epi-SCs and SKPs mixtures before transplantation inhibited the formation of neogenic hair follicles after implantation into excisional wounds in nude mice. **b** HE staining of 21-day post-grafted tissue sections with the absence (control) or presence of Perifosine or LY294002 in the graft. **c** Immunofluorescence staining of the wound tissue sections for the expression of Keratin (K) 14 and K1. Scale bars, 50 μm

To further explore reciprocal influences of the two cell types in hair follicle regeneration and identify key signals regulating the event, we performed RNA-seq analysis of Epi-SCs and SKPs after incubation in Matrigel for 24 h, and Epi-SCs and SKPs cultured separately served as controls (Fig. 3a). Bioinformatic analysis showed remarkable transcriptional changes when Epi-SCs and SKPs were cultured in mixture (Fig. 3b), suggesting profound reciprocal communications between them. KEGG analysis showed that the upregulated genes in Epi-SCs and SKPs were enriched in signals of PI3K/Akt, cancer, TNF signaling, and extracellular matrix (ECM) interaction, among them PI3K/Akt signaling pathway ranked top in Epi-SCs (Fig. 3c, d). Meanwhile, SKPs and Epi-SCs showed upregulated expression of several growth factors and cytokines, such as FGF16, CSF3, interleukin (IL)6, and oncostatin M (Tables 1 and 2), which have been known to activate PI3K [18, 19]. To get further insight into the role of PI3K/Akt signal pathway in de novo hair follicle regeneration, we performed targeted inhibition of the pathway with specific inhibitors. Either Perifosine (an Akt inhibitor) or LY294002 (a PI3K inhibitor) completely suppressed neogenic hair formation when it was added into the mixture of Epi-SCs and SKPs in Matrigel and transplanted into excisional wounds in nude mice (Fig. 4a). Histological analysis of the grafts (21 days after cell transplantation) confirmed the above finding, where hair follicles were barely detected in grafts treated with Perifosine or LY294002 (Fig. 4b). Immunofluorescence staining for the expression of Keratin 14 and Keratin 1 showed “stratified” epidermis in the grafts even after treatment with Perifosine or LY294002 (Fig. 4c). These results indicate that PI3K/Akt signal pathway plays an essential role in de novo hair follicle regeneration.

**Table 1:** Upregulated genes in Epi-SCs induced by SKPs

Gene	Description	Log_**2**_(FC)	Fold change
*Fgf21*	Fibroblast growth factor 21	12.22	4780
*Il6*	Interleukin 6	9.76	870
*Lamc3*	Laminin gamma 3	4.99	31.71
*Tnn*	Tenascin N	4.28	19.37
*Pgf*	Placental growth factor	4.14	17.67
*Thbs4*	Thrombospondin 4	3.97	15.63
*Csf3*	Colony-stimulating factor 3 (granulocyte)	3.76	13.51
*Ngf*	Nerve growth factor	3.67	12.73
*Col1a1*	Collagen, type I, alpha 1	3.65	12.51
*Osm*	Oncostatin M	3.26	9.6
*Col6a2*	Collagen, type VI, alpha 2	3.05	8.28
*Tlr2*	Toll-like receptor 2	2.94	7.67
*Fn1*	Fibronectin 1	2.91	7.50
*Vegfc*	Vascular endothelial growth factor C	2.78	6.86
*Lama4*	Laminin, alpha 4	2.42	5.36
*Fgf16*	Fibroblast growth factor 16	2.39	5.25
*Lamc2*	Laminin, gamma 2	2.31	4.97
*Col1a2*	Collagen, type I, alpha 2	2.26	4.77
*Col6a3*	Collagen, type VI, alpha 3	2.24	4.73
*Col6a1*	Collagen, type VI, alpha 1	2.23	4.69
*Tnc*	Tenascin C	2.21	4.62
*Vegfa*	Vascular endothelial growth factor A	2.10	4.30
*Lama2*	Laminin, alpha 2	1.89	3.72
*Ifnar2*	Interferon (alpha and beta) receptor 2	1.72	3.30
*Spp1*	Secreted phosphoprotein 1	1.66	3.16
*Efna1*	Ephrin A1	1.56	2.94
*Lamb3*	Laminin, beta 3	1.52	2.87
*Csf1*	Colony stimulating factor 1 (macrophage)	1.31	2.48
*Lama1*	Laminin, alpha 1	3.76	13.51
*Col4a1*	Collagen, type IV, alpha 1	1.17	2.25
*Fgf7*	Fibroblast growth factor 7	1.16	2.24
*Lamb1*	Laminin B1	1.11	2.15
*Lama3*	Laminin, alpha 3	1.04	2.06

RNA-Seq revealed upregulated (> 2-fold) genes in the category of secreted proteins in Epi-SCs after being cultured in mixture with SKPs for 24 h

**Table 2:** Upregulated genes in SKPs induced by epidermal stem cells

Gene	Description	Log_2_(FC)	Fold change
*Fgf5*	Fibroblast growth factor 5	8.53	370.00
*Osm*	Oncostatin M	8.53	370.00
*Fgf16*	Fibroblast growth factor 16	8.28	310.00
*Il6*	Interleukin 6	6.34	81.27
*Pdgfb*	Platelet derived growth factor, B polypeptide	3.87	14.67
*Csf3*	Colony stimulating factor 3 (granulocyte)	3.49	11.22
*Lama1*	Laminin, alpha 1	2.82	7.08
*Lamc2*	Laminin, gamma 2	2.79	6.89
*Lamb3*	Laminin, beta 3	2.50	5.65
*Lama5*	Laminin, alpha 5	2.38	5.21
*Lamc3*	Laminin gamma 3	2.28	4.86
*Angpt2*	Angiopoietin 2	1.89	3.72
*Lama3*	Laminin, alpha 3	1.70	3.24
*Pgf*	Placental growth factor	1.60	3.04
*Pdgfa*	Platelet-derived growth factor, alpha	1.53	2.89
*Tnc*	Tenascin C	1.49	2.81
*Efna1*	Ephrin A1	1.18	2.27
*Col6a3*	Collagen, type VI, alpha 3	1.07	2.09
*Fgf13*	Fibroblast growth factor 13	1.05	2.07

RNA-Seq revealed upregulated (> 2-fold) genes in the category of secreted proteins in SKPs after being cultured in mixture with epidermal stem cells for 24 h

## Discussion

The signaling pathway of insulin/insulin-like growth factor-1 (IGF-1)/phosphatidylinositol-3 kinase (PI3K)/Akt (also known as protein kinase B) has been involved in diverse cell activities. In mammalian cells, activation of Akt induces cell proliferation and survival, while over activated Akt signaling tends to induce cell transformation [20–23]. Previous studies suggest that Akt signaling pathway is involved with tissue regeneration; a decline in a long-term regeneration capacity of hematopoietic stem cells was found in mice with deficiency of Akt [24]; on the contrary, *Pten* loss in Lgr5 hair follicle stem cells enhanced hair follicle regeneration after wounding [11]. In this study, we show that PI3K/Akt pathway is required for Epi-SC- and SKP-based de novo hair follicle formation.

Previous studies have indicated that crosstalks between epithelial stem cells and mesenchymal cells are essential for hair follicle morphogenesis [12, 25, 26]. In postpartum humans and mice, with severe injuries to the skin, the hair follicle is barely regenerated. In recent years, hair follicle regeneration has been observed in mice in healed excisional wounds in a Wnt pathway-dependent manner [7]. Wnt signal has been considered central in hair follicle morphogenesis and regeneration. During hair follicle morphogenesis, both epidermal and dermal Wnt activations are necessary for placode formation [27, 28]. In hair follicle regeneration after wounding, FGF9 secreted by γδT cells induces dermal Wnt activation [29]. However, in many cases, hair follicle regeneration after wounding is minimal, and the mechanisms involving hair follicle de novo regeneration remain largely unclear. In our previous study, we found that the transplantation of a combination of Epi-SCs and SKPs was sufficient to generate new hair follicles [8]. Epi-SCs exhibited typical features of epidermal stem cells, while SKPs have been considered as stem cells of the dermal cells [1, 30]. In this study, we explored crosstalks between the two cell types and identified an essential role of PI3K/Akt signal pathway in de novo hair follicle regeneration. To better understand the communications between the two cell types, we used an effective 3D approach to emulate the in vivo epithelial-mesenchymal interactions [31]. We found dynamic motion and re-organization of Epi-SCs and SKPs in the matrix and demonstrated the formation of bilayer skin-like structure in vitro and after transplantation into nude mice.

It appears that Epi-SCs preserve their memory to form a stratified layer resembling the epidermis in culture and after transplantation into excisional wounds. This ability could be well preserved in the cells even after a long-term culture expansion [32]. In the present study, we found that Epi-SCs could migrate to form a distinctive epidermis-like layer over the “dermis” when cultured in mixture with SKPs in Matrigel. Upon interactions with SKPs in the graft, cells in the epidermis-like layer migrated downward to form hair follicles, a process similar to that in hair follicle morphogenesis [25]. This in vitro skin formation model facilitated us to study the reciprocal communications between the two cell types. Transcriptome analysis revealed diverse gene expressional changes upon interactions between them, which were enriched in several signal pathways, and PI3K/Akt pathway was among them. In consistence, the expression of several growth factors and cytokine that potentially activate PI3K/Akt, such as FGFs, IL6, and oncostatin M, was upregulated in both cell types. These results suggest that PI3K/Akt signaling pathway was important for the communications between the two cell populations in hair follicle regeneration. This is in consistence with our previous study, in which PI3K/Akt pathway was found to play an essential role in wounding-induced hair follicle telogen to anagen transition, and *Pten* loss in Lgr5^+^ hair follicle stem cells induced the proliferation of the stem cells leading to hair follicle regrowth [11].

Our results suggest that the communications between Epi-SCs and SKPs in hair follicle regeneration were probably mediated by molecules released by them. These molecules included growth factors, cytokines, and ECM molecules, and many of them potentially activated PI3K/Akt pathway [18, 19]. This is consistent with recent findings that platelets and mesenchymal stem cells (MSCs) promote wound healing and hair regrowth through paracrine factors, which potentially induce activation of PI3K/Akt pathway in effector cells [33–36]. Platelets are the first cell type to arrive at the site of tissue injury, where they play diverse roles through the release of various bioactive molecules [37]. A growing number of preclinical studies have shown that platelet-rich plasma (PRP), which contains a variety of growth factors [35], has multiple activities in enhancing injury repair of various tissues, such as the bone, tendon, ligament, and muscle [38, 39]. As multipotent stem cells, MSCs can differentiate into cells in the bone, cartilage, and fat [40]. Overexpression of Akt in MSCs led to enhanced cell survival after transplantation into ischemic myocardium, resulting in improved therapeutic effect on acute myocardial infarction in mice [41]. PRP was found to enhance the survival of adipose-derived stem cells (some of them are considered as MSCs) in insulin-induced adipogenesis through Akt activation, resulting in greatly increased intracytoplasmic lipid accumulation in the cells. The blockade of FGF receptor (FGFR)-1 in the cells decreased Akt phosphorylation and inhibited PRP-mediated adipogenesis [36], suggesting that FGF-induced Akt activation was an important mechanism. In agreement, exosomes in PRP was found to enhance cell survival through Akt/Bad/Bcl-2 pathway [42]. Recently, PRP has been used in several recent studies in combination with biomaterials known to enhance tissue repair. For examples, together with collagen, PRP was found to promote chondrogenic and osteogenic differentiation of adipose derived stem cells in vitro [43]. PRP was also used in combination with hyaluronic acid to repair tendons, lower-extremity wounds, posttraumatic bone exposure, and severe hidradenitis suppurativa in patients in some preliminary studies and was found to enhance the healing of these wounds [44, 45]. In another study of 10 cases, autologous fat was transplanted in combination with PRP in breast reconstruction, leading to improvement in the maintenance of fat volume [46]. These studies suggest that PRP and biomaterials support the survival and bioactivities of MSCs, and implantation of their combinations is likely to achieve a synergistic effect in tissue repair [43].

Accumulating data suggest that PRP may promote hair regrowth. Several recent studies show that PRP enhances angiogenesis in mice and promotes hair regeneration in androgenetic alopecia (AGA) patients [47–52]. In a randomized and placebo-controlled trial of 23 male patients with hair loss, local injection of PRP showed enhancement in hair regrowth [52]. These studies imply an effect of PRP on hair follicle stem cells, which are key effector cells for hair follicle growth. Indeed, in our recent study, we found that PRP, which contained various growth factors, activated quiescent hair follicle stem cells to proliferate, resulting in the transition of telogen (resting phase) to anagen (growing phase) hair follicles in mice [35]. In line with these observations, recent studies showed that transplantation of autologous cells prepared from the hair follicle could form de novo hair follicles in AGA patients [53, 54]. Based on these findings, PRP may serve as a supporting matrix for stem cells to improve their therapeutic potential in tissue repair/regeneration [53]. Taken together, based on the potential role of Akt activation as suggested in PRP therapies and our direct evidence on the role PI3K/Akt in hair follicle regeneration, the activation of PI3K/Akt pathway may serve as a therapeutic approach in hair regeneration.

## Conclusions

Taken together, our results indicate that activation of PI3K/Akt pathway is an important mechanism for epidermal and dermal cell communications which is necessary for hair follicle regeneration.

## Data Availability

The datasets used and/or analyzed during the current study are available from the corresponding author on reasonable request.

## Abbreviations

Epi-SCs: Epidermal stem cells
SKPs: Skin-derived precursors
PI3K: Phosphatidylinositol 3-kinase
AKT: Protein kinase B
DP: Dermal papilla
DMEM: Dulbecco’s modified Eagle medium
EGF: Epidermal growth factor
bFGF: Basal fibroblast growth factor
KEGG: Kyoto Encyclopedia of Genes and Genomes
EGFP: Enhanced green fluorescent protein
